# Hydrogel-Based Plasmonic Sensor Substrate for the Detection of Ethanol

**DOI:** 10.3390/s19061264

**Published:** 2019-03-13

**Authors:** Christoph Kroh, Roland Wuchrer, Nadja Steinke, Margarita Guenther, Gerald Gerlach, Thomas Härtling

**Affiliations:** 1 Institute for Solid State Electronics, Technische Universität Dresden, Mommsenstraße 15, D-01062 Dresden, Germany; mguenthe@mail.zih.tu-dresden.de (M.G.); gerald.gerlach@tu-dresden.de (G.G.); thomas.haertling@ikts.fraunhofer.de (T.H.); 2 Fraunhofer IKTS, Fraunhofer-Institute for Ceramic Technologies and Systems, Maria-Reiche-Straße 2, D-01109 Dresden, Germany; roland.wuchrer@ikts.fraunhofer.de (R.W.); nadja.steinke@ikts.fraunhofer.de (N.S.)

**Keywords:** plasmonics, ethanol sensor, hydrogel, nanostructure, hydrogel swelling, refractive index sensor, in-line sensor

## Abstract

The in-line monitoring of ethanol concentration in liquids is a crucial part of process monitoring in breweries and distilleries. Current methods are based on infrared spectroscopy, which is time-consuming and costly, making these methods unaffordable for small and middle-sized companies. To overcome these problems, we presented a small, compact, and cost-effective sensing method for the ethanol content, based on a nanostructured, plasmonically active sensor substrate. The sensor substrate is coated with an ethanol-sensitive hydrogel, based on polyacrylamide and bisacrylamide, which induces a change in the refractive index of the substrate surface. The swelling and shrinking of such hydrogels offer a means to measure the ethanol content in liquids, which can be determined in a simple transmittance setup. In our study, we demonstrated the capability of the sensor principle for the detection of ethanol content ranging from 0 to 30 vol% ethanol. Furthermore, we determined the response time of the sensor substrate to be 5.2 min, which shows an improvement by a factor of four compared to other hydrogel-based sensing methods. Finally, initial results for the sensor’s lifetime are presented.

## 1. Introduction

Efficient and low-cost sensors for the detection of chemical parameters in liquids are facing an ever-increasing demand in many different application fields, such as medical care or the food industry. Ethanol content in particular is one major parameter for process monitoring in breweries or distilleries.

Current detection methods for ethanol are based on the indirect calculation of ethanol content by oscillating density meters [1], or on a spectral determination by means of infrared spectroscopy [2,3]. Such systems require a laboratory environment and are very time-consuming and costly, which makes them unaffordable for many small and middle-sized breweries. In comparison, mobile laboratory devices like hydrometers have poor resolution and a strong temperature dependency [4], which makes them unsuitable for an accurate monitoring of the ethanol content over a time period of 7–10 days—the typical time span of the fermentation process. Furthermore, it is preferable to detect the ethanol content directly inside the brewing tanks. This demands a small, compact, and robust sensor device, for a continuous and simultaneous in-line monitoring of parameter changes in a complex medium.

One approach to measuring the ethanol content is based on the use of ethanol sensitive hydrogels. Hydrogels are hydrophilic, non-soluble, cross-linked polymers that show a reversible decrease in volume as a response to a chemical parameter change (in our case increasing ethanol content) in their environment. Thus, interrogating the swelling–shrinking state of the hydrogel allows the ethanol content to be deduced. The volume change can be detected, for instance, by means of a pressure sensor [5,6,7,8], which demands rather thick hydrogel sensor layers ranging from 50 to 330 μm in the dried state [5,6,7] and up to 500 µm in the swollen state [8]. In [9], a quartz crystal microbalance (QCM) was coated with 0.4 µm and 0.8 µm thick polymer layers, respectively. Due to the dependence between the layer thickness and response behavior [10], the equilibrium state in these systems is typically reached only after more than 20 min [5,6,8]. One way to improve the response time of hydrogel-based sensors is to interrogate the ethanol-induced swelling state optically. To this end, we combined an ethanol-sensitive hydrogel with a nanostructured gold sensor substrate. This nanostructured metal surface supports localized plasmon oscillations [11,12], which can be excited in a simple transmittance setup. The plasmon oscillations are sensitive to refractive index changes in the vicinity of the metal substrate, which in turn appear as spectral changes of the transmittance spectrum [13,14]. During the swelling and shrinking of the hydrogel induced by changing ethanol concentrations, water diffuses in or out of the gel, which affects the polymer chain–water ratio. This leads to a change in the effective refractive index on the metal–polymer boundary, and subsequently to spectral changes in the transmittance spectrum of the sensor substrate. The sensitivity of the substrates is restricted to a layer in the range of a couple of 100 nm thickness perpendicular to the surface only, which allows the lowering of the hydrogel layer thickness to <1 µm. This will result in a shorter diffusion time of the analyte through the hydrogel layer, a faster establishment of the volume change equilibrium, and, consequently, a faster response time compared to other hydrogel-based sensor systems. 

Ethanol-dependent swelling has already been reported for different hydrogel systems and sensor concepts [15,16]. In this work, the combination of a polyacrylamide–bisacrylamide-based ethanol-sensitive hydrogel and a nanostructured gold sensor substrate is introduced for the first time. The overall aim of this work is to develop a robust hydrogel-based sensor for the in-line measurement of ethanol, with an operational lifetime of more than 10 days. Therefore, we present our nanostructured sensor substrate, as well as the optical detection of different hydrogel swelling states induced by various ethanol contents. Furthermore, we report initial results concerning the long-term stability of this sensor substrate, which provide the first data for the desired sensor application in the brewing industry.

## 2. Materials and Methods

### 2.1. Materials

The substrate foil cycloolefin polymer (COP) was purchased from Microfluidic ChipShop (Jena, Germany). For the nanoimprint lithography, the photoresist SU-8 was obtained from micro resist technology (Berlin, Germany), the polydimethylsiloxane (PDMS) RTV-615 was purchased from KVD (Bad Wimpfen, Germany), and the silicon master containing the nanostructure was fabricated by Eulitha AG (Kirchdorf, Switzerland). For the surface functionalization of the sensor substrates, N,N′–bis(acryloyl)cystamine (BAC) was purchased from Alfa Aesar. The monomer acrylamide (Aam) and the photoinitiator lithium phenyl–2,4,6–trimethylbenzoylphosphinate were purchased from Sigma Aldrich. The cross-linker N,N′–methylen–bis–acrylamide (BIS) was obtained from Carl Roth GmbH + Co. KG (Karlsruhe, Germany).

### 2.2. Fabrication of the Sensor Substrate

**Figure 1:**
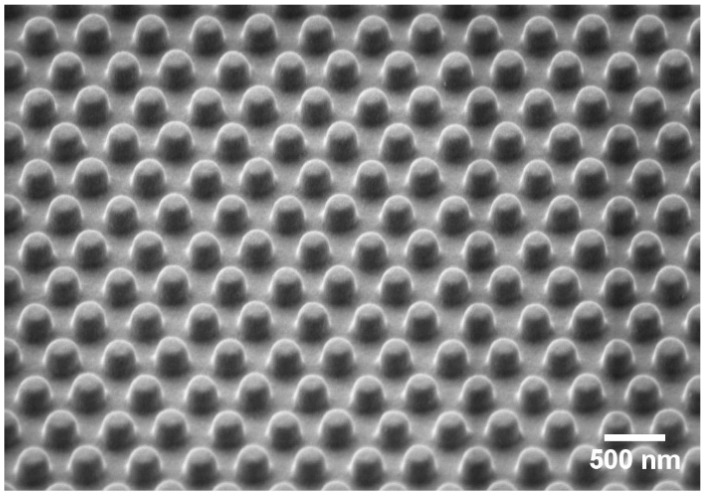
Scanning electron microscope image (SEM) of the nanostructured gold sensor substrate.

The plasmonic sensor substrate consisted of a 3 × 3 mm^2^ nanopillar array in a hexagonal lattice, fabricated by means of nanoimprint lithography, according to Yang et al. [11]. A silicon master fabricated by means of UV lithography was used to transfer a negative of the nanostructure into a PDMS mold. The PDMS was imprinted on a UV-curable SU-8 photoresist, which was uniformly spread on the substrate carrier, using the microcontact printer µCP 4.1 (GeSiM mbH, Großerkmannsdorf, Germany). To enable a homogeneous distribution of the photoresist, the wafer was heated to a temperature of 95 °C before imprinting. A 4 inch COP-foil wafer with a thickness of 188 µm was used as a substrate carrier. Subsequently, the nanopillars were cured with UV radiation, and the PDMS mold was removed. The nanopillars had a diameter of 230 nm, a center-to-center distance of 450 nm, and a height of 150 nm (Figure 1). Finally, the substrates were coated with a 2 nm thick chromium adhesive layer, and a 40 nm gold layer for surface plasmon activation. The wafer was cut into square pieces with a side length of 10 mm.

### 2.3. Hydrogel Synthesis and Immobilization on the Sensor Substrate

For the hydrogel immobilization, the sensor substrates were rinsed with ethanol and treated with UV/ozone (UV/ozone ProCleaner, NanoAndMore GmbH, Wetzlar, Germany) for 20 min. For a covalent binding of the hydrogel, the sensor substrates were treated with BAC (0.4 mmol) dissolved in dimethyl sulfoxide (DMSO). 

The hydrogels were synthesized according to the protocol of Erfkamp et al. [8]. Firstly, 10 vol% (0.32 mmol) of acrylamide (Aam) and 0.44 mol% (1.39 µmol) of bisacrylamide (BIS) were diluted in distilled water and degassed in a nitrogen atmosphere for 5 min. Subsequently, 1 vol% (32 µmol) of the photo initiator lithium phenyl–2,4,6–trimethylbenzoylphosphinate (LAP) were added to the stock solution and mixed for 10 min in a nitrogen atmosphere. Each sensor substrate was coated in 10 µL of the stock solution. Polymerization was finally carried out with UV light (wavelength range 320–500 nm) for 2 min. To remove residues of the monomer and the photo initiator, the sensor substrates were washed in distilled water for 48 h. After the synthesis, the sensor substrates containing the hydrogel were placed alternately in pure ethanol and distilled water five times for conditioning.

### 2.4. Studies of the Free Swelling of the Hydrogel

For the investigation of the free swelling, 5 mm squares of the polyacrylamide (PAM) hydrogel were synthesized. After synthesis and conditioning, the initial length *l*_0_ of the swollen hydrogel samples was determined by means of microscopy, with five-fold averaging. Subsequently, the shrinking of the gels was induced by incubation in a 50 vol% ethanol solution for two hours. After the determination of the shrunken length *l* of the gels, the solution was changed back to distilled water. This process was repeated for several cycles. From the results, the swelling degree *S* of the hydrogel was calculated as the relative change in length as follows [17]:

(1) $$S=\frac{l}{l_{0}}\;.$$

### 2.5. Plasmonic Transducer and Sensor Setup 

**Figure 2:**
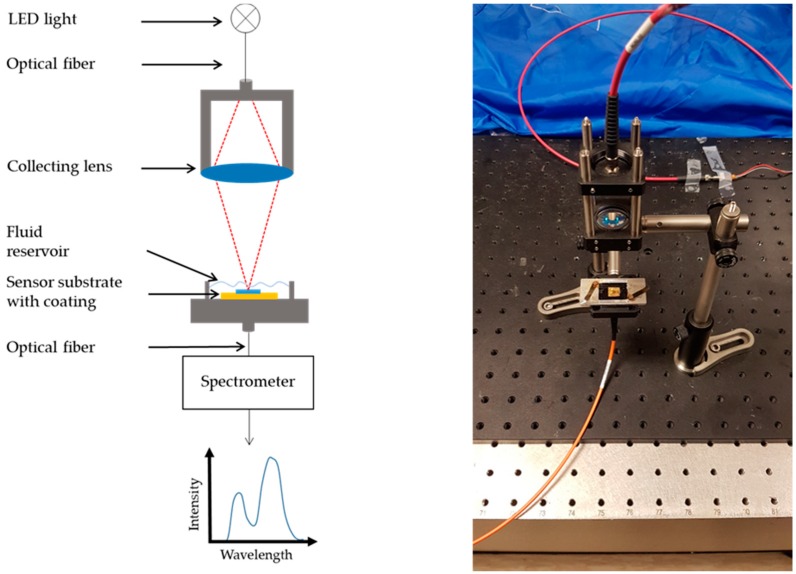
Schematic representation and picture of the sensor setup to record the transmittance spectra of the sensor substrates.

The sensors consisted of a nanostructured gold substrate, which is well known to support localized surface plasmon oscillations [11,12,13]. These collective electron oscillations can be excited in the nanostructured metal layer with normal incident light without additional optics. They cause a local electromagnetic field enhancement on the substrate surface within a range of a couple of 100 nm distance [14,18]. As described in many previous studies, these plasmon oscillations also lead to absorption and scattering of the incident light, which cause a local dip in the transmittance spectrum of the sensor substrate [13,14]. This dip, representing the plasmon oscillation resonance wavelength, subsequently results from a superposition of the incident LED spectrum and the extinction spectrum of the nanostructure, and shifts linearly with the refractive index in the vicinity of the sensor substrate [19,20]. This shift serves as the sensor signal and is independent of the intensity and initial position of the spectrum. Thus, such structures can be used as sensor elements for the detection of refractive index changes, and as a transducer element for hydrogels. The optical interrogation of the sensor substrate was accomplished with a simple home-built transmittance evaluation system. The substrates were bonded on an optical holder with PDMS and were irradiated with an LED (central wavelength 875 nm, spot size 1 mm) in the spectral window between 750 and 1000 nm. The light transmitted through the sensor substrate was collected with an optical fiber and directed to a spectrometer (iHR550, Horiba Jobin Yvon GmbH, Unterhaching, Germany), which recorded the transmittance spectra with a resolution of 0.03 nm (diffraction grating with 1200 slits). After smoothing by means of an FFT filter, the resonance wavelength was calculated from a Gaussian fit of each transmittance spectrum. Figure 2 shows a schematic representation and a picture of the sensor setup.

## 3. Results and Discussion

### 3.1. Characterization of the Sensor Substrate

As a first step, the quality of the sensor substrates, including the spectral position of the resonance wavelength and the refractive index sensitivity, was determined. In order to achieve this, the sensor substrates were placed in aqueous solutions with different ethylene glycol content (see Table 1) to analyze the refractive index sensitivity of the sensor substrate. The reference measurement was carried out by means of a refractometer (DR201-95, Krüss Optronic, Hamburg, Germany), with a measurement uncertainty of 0.0003 RIU (refractive index unit).

A droplet (20 µL) of each solution was placed on the sensor substrate and the respective transmittance spectrum was measured. 

**Figure 3:**
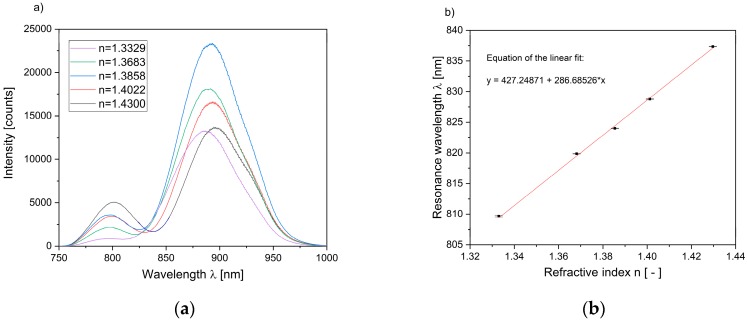
Interrogation of the sensitivity of the sensor substrates (**a**) Transmittance spectra of sensor substrates covered with water–ethylene glycol solutions of different refractive index n; (**b**) Linear shift of the resonance wavelength with increasing refractive index resulting in a sensitivity of 287 nm RIU^−1^.

Subsequently, the refractive index sensitivity of the substrate was determined from the slope of a linear fit of the resonance wavelength. Figure 3a shows the transmittance spectra for the different H_2_O–EG solutions, while in Figure 3b the correlation between the refractive index and plasmon resonance wavelength is depicted. The spectral position of the resonance wavelength in water was determined by means of a Gaussian fit (see Section 2.5) to 810 nm. The spectra show a red-shift of the resonance wavelength with an increasing refractive index, and the sensitivity was calculated to 287 nm per RIU, which implies a resolution of the refractive index in the order of 10^−4^. The plotted uncertainty bars, ranging from 0.04 nm to 0.16 nm, originated from the standard deviation of a five-fold averaging, in addition to the uncertainty of the Gaussian fit. The changes in the intensities of the spectra are caused by performance fluctuations and warming of the LED. Furthermore, absorption depends on the refractive index, which causes additional changes to the intensity. Hence, the resonance wavelength is independent of the intensity; such changes have no impact on the sensor signal and can consequently be disregarded.

### 3.2. Optical Interrogation of Hydrogel Swelling States Induced by Ethanol

#### 3.2.1. Reversible Free Swelling of the Hydrogel

Hydrogels are well known for their reversible swelling process, which makes them interesting candidates for sensor materials. The swelling and shrinking mechanisms induced by ethanol were studied in [21,22]. After the addition of ethanol, the PAM hydrogel deswells. This can be explained by the influence of the alcohol on the water–polymer systems, whereby the free energy of the polymer–polymer contact increases, and the polymer chains attract each other. This causes a diffusion of water out of the gel and, subsequently, an increase of the refractive index inside the system. By decreasing the amount of ethanol, the free energy decreases and the gel swells back to its original state [21]. A further approach to describing the swelling mechanism of ethanol-sensitive hydrogels is based on the positive viscosity coefficient of ethanol, which causes stronger interactions inside the gel. These interactions again induce an attraction of the polymer chains, and a shrinking of the gel with increasing ethanol content [22]. 

**Figure 4:**
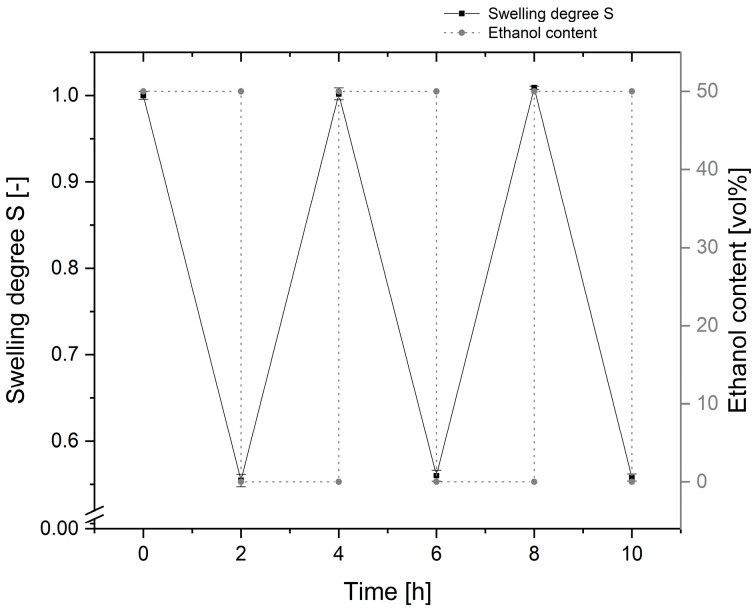
Reversible hydrogel swelling of the polyacrylamide (PAM) hydrogel versus ethanol content of the solution. The swelling degree S shows a decrease of about 45% between distilled water and a 50 vol% ethanol solution.

Figure 4 shows the reversible free swelling of the PAM hydrogel over multiple cycles. The swelling degree of the hydrogel was determined according to the methods outlined in Section 2.4. The uncertainty bars originated from the standard deviation of the applied five-fold averaging. 

After a change from 0 to 50 vol% ethanol content, the hydrogel showed a reversible shrinking of about 45% of its size over multiple cycles. This is in accordance with previous studies of the PAM-based hydrogel [8], and shows the suitability of such gels as a sensor material.

#### 3.2.2. Optical Detection of Different Hydrogel Swelling States

**Figure 5:**
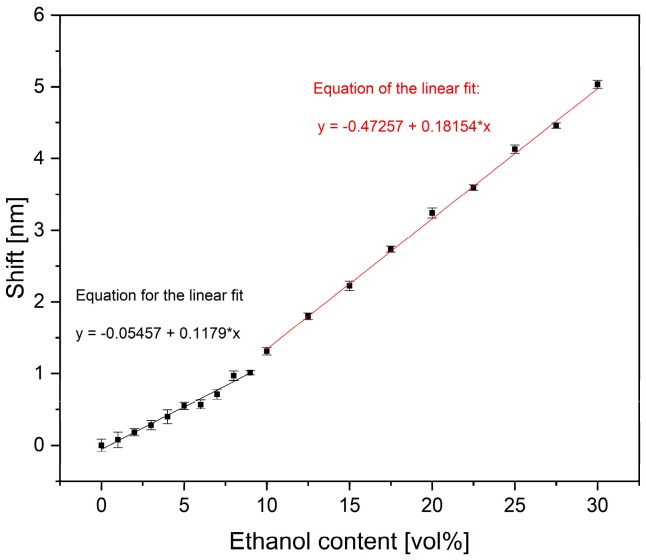
Shift of the resonance wavelength with increasing ethanol content due to the deswelling of the PAM hydrogel showing a linear behavior of the refractive index change.

The sensor substrates were coated with the PAM hydrogel and conditioned with water and ethanol solutions. To attain different swelling states, the sensor substrates were placed on the holder (see Figure 2), bonded with PDMS, and the fluid reservoir was filled with water containing different levels of ethanol content. After a 15 min immersion in each solution, the transmittance spectrum of the sensor substrate was taken by applying five-fold averaging. The resonance wavelength of the respective spectrum was determined with a Gaussian fit, and its average position was plotted over the respective ethanol content. For the optical monitoring of the swelling and shrinking, a hydrogel layer with a thickness of about 20 µm was used. Figure 5 presents the position of the resonance wavelengths for an ethanol content between 0 and 30 vol%. This position shows a red-shift with increasing ethanol content, which corresponds to an increasing effective refractive index of the gel. The plotted uncertainty bars originated from the standard deviation of the five-fold averaging, in addition to the uncertainty of the Gaussian fit. 

The shrinking behavior of the hydrogel indicates a section-wise linear increase of the refractive index inside the hydrogel between 0 and 30 vol% ethanol content. We observed a clear shift of the resonance wavelength between 0 and 10 vol%, with an interval of 1 vol%. The slope of the shown data, representing the sensitivity of the sensor device, was determined as 0.118 nm per vol% ethanol. With respect to the resolution of the spectrometer (0.03 nm), this would lead to a theoretical resolution of 0.3 vol% of ethanol and implies the suitability of the sensor substrate as a monitoring device in the brewing process. For higher ethanol concentrations, we observed a sensitivity of 0.182 nm per vol% ethanol, which would lead to a theoretical resolution of <0.2. 

We attributed the different sensitivities to a delayed starting behavior of the hydrogel swelling in low ethanol concentrations. This behavior, typical of the hydrogel, was described in previous works [8] and will require separate calibration curves depending on the desired application.

#### 3.2.3. Swelling Kinetics and Response Time

**Figure 6:**
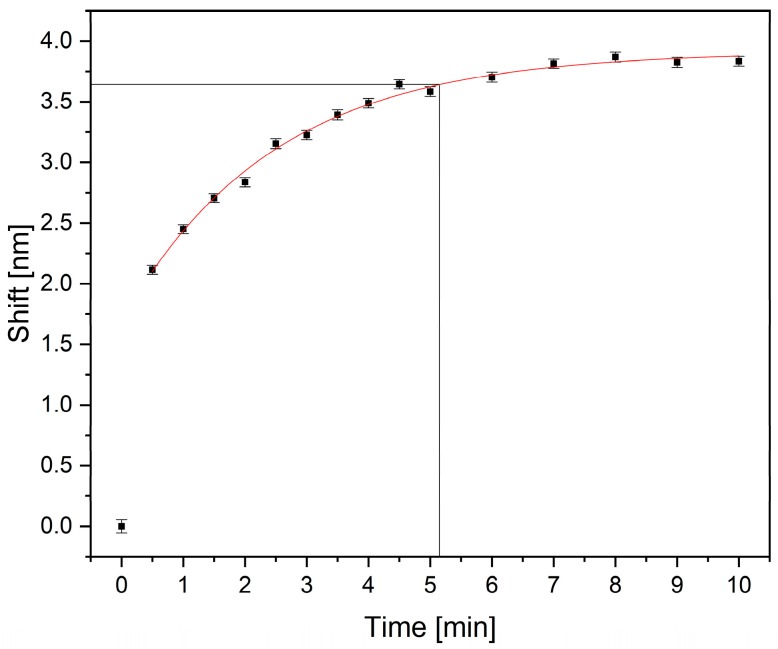
Time-resolved response of the sensor substrate covered with PAM hydrogel after a change from water to a 20 vol% ethanol solution.

In addition to the swelling behavior at different ethanol contents, a fast in-situ response is a crucial requirement of sensor devices. To determine the response time of the sensor material, the fluid reservoir was filled with water and the hydrogel was immersed for 30 min. Afterwards, we investigated the hydrogel shrinking in a 20 vol% ethanol solution over 10 min. Figure 6 shows the time-resolved shift of the resonance wavelength by means of the in-situ shrinking behavior of the PAM hydrogel, induced by a change in the ethanol content. The plotted uncertainty bars originated from the standard deviation of the Gaussian fit, which was used to determine the resonance wavelength.

The results show an exponential increase of the resonance wavelength. It can clearly be seen that the gel reacts immediately upon ethanol contact, and more than 50% of the shift is reached after 0.5 min. After 5.2 min, 95% of the steady-state value is reached, which shows an improvement by a factor of four compared to other hydrogel-based sensor systems [5,6,8]. This improvement is caused by the much thinner hydrogel layer, which in turn leads to a lower diffusion time of the fluid out of the gel and, subsequently, to a faster response to changing ethanol content.

#### 3.2.4. Long-Term Stability of the Sensor Substrate

For use as an in-line sensing platform in the brewing process, a lifetime of 7–10 days is required for the sensor substrate. To initially investigate its long-term stability, the substrate was placed on the holder for 10 days and immersed in distilled water. Periodically, the liquid was changed to a 50 vol% ethanol solution, and the transmittance spectra were determined by a five-fold averaging both after each deswelling in ethanol and each distilled water immersion with constant swelling times. Afterwards, the ethanol solution was replaced again by distilled water. The resonance wavelengths were determined with a Gaussian fit. 

**Figure 7:**
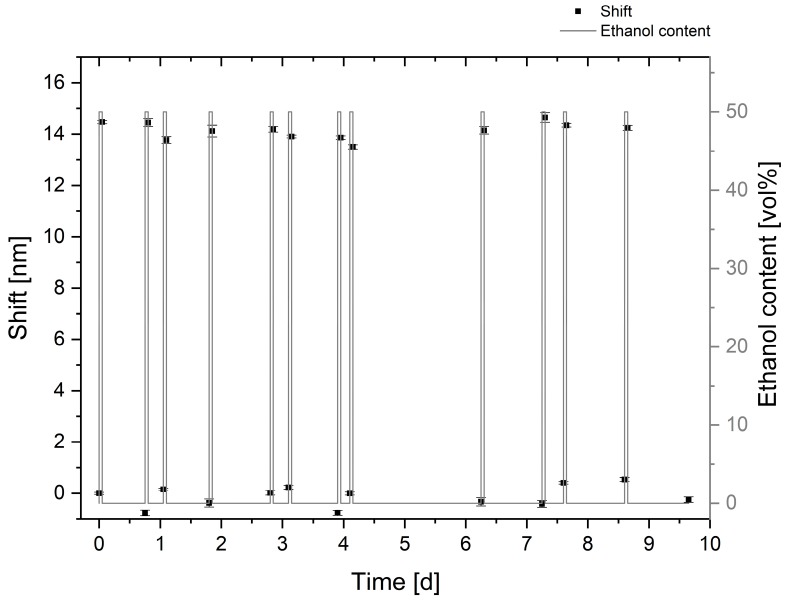
Signal stability of the resonance shift of the sensor substrate, coated with the PAM hydrogel, investigated over 10 days by a periodically induced deswelling in a 50 vol% ethanol solution.

Figure 7 presents the initial results for the long-term stability of the sensing material. The plotted error bars originated from the standard deviation of the five-fold averaging and of the uncertainty of the Gaussian fit. 

The results show a shift in the resonance wavelength, induced by the shrinking of the hydrogel in ethanol of about 14 nm, with no significant change over 10 days. Furthermore, the sensor substrate and the hydrogel were stable and no effects of ageing and delamination could be observed. 

The deviations, we assume, could be caused by temperature variations during the experiment duration and the evaporation of the ethanol. However, this data proves the suitability of the surface functionalization for a bonding of the PAM hydrogel on the gold sensor substrates. 

## 4. Conclusions and Outlook

In this paper, we reported on the suitability of an optical detection method for the determination of the ethanol concentration in aqueous solutions. We applied a nanostructured gold sensor substrate supporting localized surface plasmons as an optical transducer for an ethanol-sensitive hydrogel, to enable the detection of refractive index changes occurring during the swelling and deswelling of the gel. The combination of an optical transducer element and the PAM-based ethanol-sensitive hydrogel was studied for the first time. 

The results showed a linear red-shift of the resonance wavelength in the nanostructure transmittance spectra, with increasing ethanol content due to the deswelling of the hydrogel, and a subsequently increasing refractive index on the transducer surface. It was shown that this shift resulted in a theoretical resolution of the ethanol content of about 0.4 vol%. Furthermore, we showed an improvement in the response time in comparison to other hydrogel-based sensor systems by a factor of four, which led to a response time of about 5.2 min. Additionally, we showed a sensor lifetime and a signal stability of more than 10 days, which would be sufficient for possible application in the brewing process. 

However, it is still possible to improve the resolution of the sensor substrate, e.g., by modifying the hydrogel. As shown in [8], a decrease in the monomer or cross-linker content would lead to an enhanced deswelling of the gel, and subsequently to an increase of the sensitivity. However, this would cause a reduction in the mechanical stability of the gel, whereby an optimum between the latter and the sensitivity must be found. Another possibility to improve the sensitivity is the increase of the sensitivity of the optical sensor substrate itself. 

Furthermore, the determined response time implies the suitability of the sensor substrate as an in-line process monitoring tool in the brewing process, where measurement intervals of 30 min are common. A further improvement in the response time can be obtained with even thinner hydrogel layers. For a plasmonic readout, a hydrogel layer thicknesses of around 1 µm would be sufficient, which can be reached by the uniform or microstructured imprinting of the hydrogels. Future work will focus on the implementation of even thinner hydrogel layers by way of microstructuring, the optimization of the hydrogel sensitivity, and the cross-sensitivities of the gel to other parameters.

Nevertheless, the linear behavior, the high sensitivity, and the short response time of the plasmonic sensor system indicate its suitability as a platform for the in-line process monitoring of alcoholic fermentation in breweries.

## Figures and Tables

**Table 1:** Water–ethylene glycol solutions for the calibration measurement of the refractive index.

Solution	Refractive Index
100 v% H_2_O	1.3329
66 v% H_2_O, 33 v% EG	1.3683
50 v% H_2_O, 50 v% EG	1.3858
33 v% H_2_O, 66 v% EG	1.4022
100 v% EG	1.4300
